# Spontaneous Adrenal Hemorrhage in a Pregnant Woman With Glucocorticoid Resistance Syndrome

**DOI:** 10.1210/jcemcr/luae052

**Published:** 2024-04-17

**Authors:** Varsha Jagtap, Anurag Lila, Manjiri Karlekar, Vijaya Sarathi, Tushar Bandgar

**Affiliations:** Consultant Endocrinologist, Deenanath Mangeshkar Hospital, Pune 411004, India; Department of Endocrinology, Seth G.S. Medical college and KEM Hospital, Mumbai 400012, India; Department of Endocrinology, Seth G.S. Medical college and KEM Hospital, Mumbai 400012, India; Department of Endocrinology, Vydehi Institute of Medical Sciences and Research Centre, Bangalore 560066, India; Department of Endocrinology, Seth G.S. Medical college and KEM Hospital, Mumbai 400012, India

**Keywords:** glucocorticoid resistance syndrome, *NR3C1*, adrenal hemorrhage, hypokalemic paralysis

## Abstract

Glucocorticoid resistance syndrome is a rare disorder with no genetically proven cases reported from India; in addition, there are no descriptions available regarding its management during pregnancy. A 27-year-old woman, hypertensive since the age of 17 years, presented with hypokalemic paresis. She reported regular menses and acne. On investigation, she had elevated serum cortisol that remained unsuppressed after a low-dose dexamethasone suppression test. Genetic analysis revealed a novel, homozygous missense variant in exon 5 of the *NR3C1* gene confirming glucocorticoid resistance syndrome. She was managed with oral dexamethasone followed by tapering of antihypertensive drugs. A year later, she conceived with assisted reproductive techniques when dexamethasone was replaced with prednisolone, necessitating the reintroduction of antihypertensive drugs to maintain normotension and potassium supplements to manage hypokalemia. She presented with acute abdomen at 36 weeks of gestation; evaluation revealed right adrenal hemorrhage, which was managed conservatively. Postpartum, the right adrenal lesion reduced in size and an underlying right adrenal myelolipoma was unveiled.

## Introduction

Glucocorticoid resistance syndrome (GCRS) is a disease resulting from defective signaling in the glucocorticoid receptor pathway. GCRS has a variable presentation that includes hypertension, hypokalemia, hyperandrogenism, and bilateral adrenal hyperplasia [1, 2]. GCRS is characterized by biochemical hypercortisolism but lacks clinical features of Cushing syndrome (CS) because of resistance at the level of glucocorticoid receptors. Although GCRS is a rare cause of hypertension, recognition of the condition is essential for optimal blood pressure (BP) management. Though a patient with GCRS diagnosed based on clinical and hormonal evaluation has been reported previously from India, no genetically proven case of GCRS has been reported from India [3]. There are limited data regarding the management of GCRS during pregnancy. Here, we report the first Indian patient with genetically proven GCRS and unique challenges in her management during pregnancy.

## Case Presentation

A 27-year-old woman presented with quadriparesis following an episode of acute gastroenteritis. On investigation, she was found to have hypokalemia (serum potassium: 2.1 mmol/L; normal reference range: 3.5-5.5 mmol/L), and her quadriparesis resolved with potassium supplementation. She had been diagnosed with hypertension at age 17 years and was managed with oral amlodipine (10 mg per day) and telmisartan (40 mg per day). She also had a previous history of hypokalemia (1.8 mmol/L) diagnosed at age 22 years when evaluated for muscle cramps, for which she received oral potassium supplements for a few months. She had regular menses but had been complaining about facial hirsutism and acne resistant to topical therapy since the age of 14 years. On physical examination, she was conscious, oriented in time place and person; weighed 66 kg, with a body mass index of 23.67 kg/m^2^; and had a BP of 136/90 mm Hg. She exhibited no signs of CS or muscle weakness.

One of her younger sisters had a history of hirsutism and acne from the age of 15 years. On evaluation at 24 years of age, she was also found to be hypertensive (BP: 140/90 mm Hg). Her father had hypertension from the age of 40 years, well-controlled with oral telmisartan (40 mg per day). Her mother and another younger sister were normotensive with no features of hyperandrogenism or menstrual abnormalities. On examination, the proband exhibited mild hirsutism (modified Ferriman Gallwey score: 10) and clitoral enlargement (8 × 5 mm) but showed no posterior labial fusion (anogenital ratio: 0.45).

## Diagnostic Assessment

Laboratory evaluation was conducted after obtaining informed consent from the subject, revealing hypokalemia, hyperandrogenemia, and corticotropin (ACTH)-dependent endogenous hypercortisolism (Table 1). Considering the ACTH-dependent hypercortisolism, the absence of cushingoid features, and low renin hypertension with hyperandrogenism, a provisional diagnosis of GCRS was made. Clinical exome sequencing revealed a novel homozygous missense variant, p. Leu545Ser, in exon 5 of *NR3C1* gene. In silico predictions indicated that the variant is possibly damaging according to PolyPhen-2 (HumDiv) and classified as damaging by Sorting Intolerant From Tolerant, likelihood ratio test, and MutationTaster2. This variant is not reported in gnomAD and 1000 Genomes databases, whereas the wild-type codon is conserved across the species. The variant was confirmed by Sanger sequencing. Although both parents were heterozygous for the variant, the affected sister was homozygous. An evaluation of the affected sister at age 24 years revealed hypokalemia (potassium: 3.3 mmol/L), an elevated morning serum cortisol of 68.3 µg/dL (1884.1 nmol/L) (normal reference range: 5-25 µg/dL; 138-690 nmol/L), and a total testosterone level of 1.08 ng/mL (3.74 nmol/L) (normal reference range: 0.2-0.8 ng/mL; 0.7-2.7 nmol/L). The serum cortisol was unsuppressed (54.6 µg/dL, 1506.2 nmol/L) after an overnight dexamethasone suppression test (normal reference range: <1.8 µg/dL; <50 nmol/L).

**Table 1.** Laboratory investigations of the proband

Laboratory test	At diagnosis	At the last follow-up (on dexamethasone 4 mg per day)	Reference range
Serum potassium	2.1 mEq/L (2.1 mmol/L)	4.9 mEq/L (4.9 mmol/L)	3.5-5.2 mEq/L (3.5-5.2 mmol/L)
Hemoglobin	13.1 g/dL (8.13 mmol/L)	12.6 g/dL (7.82 mmol/L)	12-16 g/dL (7.4-9.9 mmol/L)
Leukocyte count (cells/µL)	8600 cells/µL (8.6 cells ×10^9^ L)	7520 cells/µL (7.5 cells ×10^9^ L)	4000-11000 cells/µL (4.0-11.0 cells ×10^9^ L)
Neutrophils (%)	58%	61%	40%-60%
Lymphocytes (%)	35%	34%	20%-40%
Eosinophils (%)	3.2%	2.9%	1%-4%
Fasting plasma glucose	89 mg/dL (4.94 mmol/L)	86 mg/dL (4.77 mmol/L)	70-100 mg/dL (3.9-5.6 mmol/L)
Serum total cholesterol	155.9 mg/dL (4.03 mmol/L)	148.2 mg/dL (3.83 mmol/L)	<200 mg/dL L (<5.2 mmol/L)
Serum triglycerides	88.1 mg/dL (0.99 mmol/L)	85.3 mg/dL (0.96 mmol/L)	<150 mg/dL (<1.69 mmol/L)
Serum LDL-cholesterol	96.4 mg/dL (2.49 mmol/L)	92.1 mg/dL (2.38 mmol/L)	<100 mg/dL (< 2.6 mmol/L)
Serum HDL-cholesterol	42.3 mg/dL (1.09 mmol/L)	40.2 mg/dL (1.03 mmol/L)	>50 mg/dL (>1.3 mmol/L)
Plasma ACTH	65 pg/mL (14.31 pmol/L)	—	10-60 (2.2-13.2 pmol/L)
Plasma aldosterone	3.05 ng/dL (0.08 nmol/L)	4.99 ng/dL (0.14 nmol/L)	10-45 ng/dL (0.28-1.2 nmol/L)
Serum cortisol	98.4 µg/dL (2715.5 nmol/L)	8.9 µg/dL (245.5 nmol/L)	5-25 µg/dL (138-690 nmol/L)
Serum 11-deoxycortisol (ng/mL)	11.2 ng/mL (32.3 nmol/L)	1.14 ng/mL (3.3 nmol/L)	0.5-3.0 ng/mL (1.4-8.65 nmol/L)
Serum 11-deoxycorticosterone	1.03 ng/mL (3.12 nmol/L)	0.24 ng/mL (0.73 nmol/L)	0.02-0.15 ng/mL (0.06-0.45 nmol/L)
Serum corticosterone	15.8 ng/mL (46.1 nmol/L)	3.84 ng/mL (10.96 nmol/L)	1-20 ng/mL (3-57.6 nmol/L)
Serum 17-hydroxyprogesterone	4.24 ng/mL (12.8 nmol/L)	1.15 ng/mL (3.48 nmol/L)	0.2-2.2 ng/mL (0.6-6.6 nmol/L)
Serum androstenedione	6.48 ng/mL (22.6 nmol/L)	0.76 ng/mL (2.6 nmol/L)	0.18-2.6 ng/mL (0.62-9.0 nmol/L)
ONDST cortisol	71.8 µg/dL (1980.6 nmol/L)	—	<1.8 µg/dL (<50 nmol/L)
Serum total testosterone	1.62 ng/mL (5.6 nmol/L)	0.25 ng/mL (0.86 nmol/L)	0.2-0.8 ng/mL (0.7-2.7 nmol/L)
Serum DHEAS	738.1 µg/dL (20.0 µmol/L)	94.4 µg/dL (2.56 µmol/L)	95.8-511.7 µg/dL (2.6-13.8 µmol/L)
Progesterone	0.56 ng/mL (1.8 nmol/L)	0.43 ng/mL (1.3 nmol/L)	<0.89 ng/mL (2.3 nmol/L)
Plasma renin activity	0.09 ng/mL/hr 0.09 µg/L/hr	—	0.6-4.3 ng/mL/hr 0.6-4.3 µg/L/hr
Direct renin concentration	—	28.3 µIU/mL	4.4-46.1 µIU/mL

Abbreviations: DHEAS, dehydroepiandrosterone sulphate; ONDST, 1-mg overnight dexamethasone suppression test.

## Treatment

**Figure 1.**
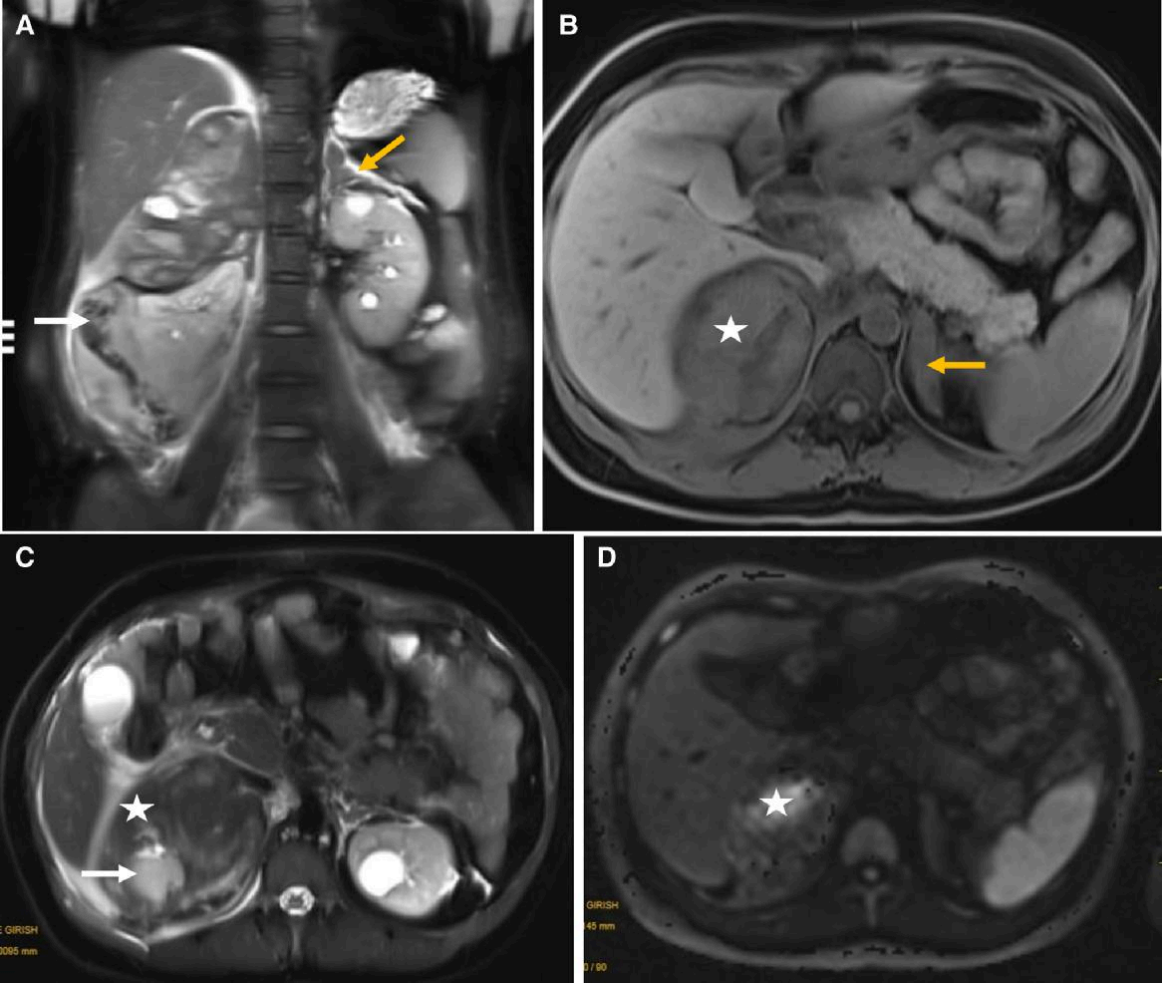
Magnetic resonance imaging showing a well-defined lesion (8.7 × 8.2 × 11 cm) in the right suprarenal area that was heterogeneously hyper-isointense on T2-weighted half Fourier single-shot turbo spin-echo coronal image (A), hyperintense with no fat suppression on axial T1-weighted fat saturation sequences (B) with breach in the posterior capsule with fluid/clots in the right posterior perinephric and pararenal space suggestive of right adrenal hemorrhage (white arrows) (A,C). Corresponding patchy areas of diffusion restriction representing acute hemorrhage (white star; B-D) with adjacent subacute hemorrhage and clots. The left adrenal gland is homogenously iso-hypointense and enlarged (4.4 × 1.2 × 3.1 cm), suggesting left adrenal hyperplasia (yellow arrows).

After confirming the diagnosis of GCRS, she was initiated on oral dexamethasone at a dosage of 3 to 4 mg per day. Antihypertensives and potassium supplements were gradually tapered. During follow-up, serum potassium levels ranged from 3.4 to 4.9 mmol/L, morning cortisol levels ranged from 8.7 to 26.2 µg/dL (240-722 nmol/L), and direct renin concentrations ranged from 4.5 to 32.5 µIU/mL (normal reference range: 4.4-46.1 µIU/mL). A year later, she conceived with in vitro fertilization. After confirmation of pregnancy at 6 weeks of gestation, dexamethasone was discontinued and prednisolone was initiated at a dosage of 20 mg per day. Because of an increase in BP (144/92 mm Hg), recurrence of hypokalemia (serum potassium: 3.2 mmol/L) and increase in serum morning cortisol (from prepregnancy level of 12.8 µg/dL [353.1 nmol/L] to 28.1 µg/dL [755.1 nmol/L]) at 10 weeks of gestation), oral amlodipine (10 mg per day), labetalol (200 mg per day), and potassium supplements (60 mEq per day) were added. Her BP was well controlled (ranging from 110/70 mm Hg to 130/84 mm Hg) on this regimen until the last antenatal visit at 34 weeks. However, at 36 weeks of gestation, she presented with acute abdominal pain. An ultrasonogram revealed a right adrenal mass suggestive of hemorrhage, later confirmed on magnetic resonance imaging (Fig. 1). She was started on intravenous betamethasone (6 mg 4 times per day). Twenty-four hours later, she underwent a lower segment cesarean section and delivered a preterm male neonate who required care in a level 2 neonatal intensive care unit for a week. Postpartum, the patient was transitioned to intravenous dexamethasone for 2 days and subsequently to oral dexamethasone (2 mg per day), ensuring effective BP control and facilitating the gradual tapering off of antihypertensive drugs and potassium supplements.

## Outcome and Follow-up

**Figure 2.**
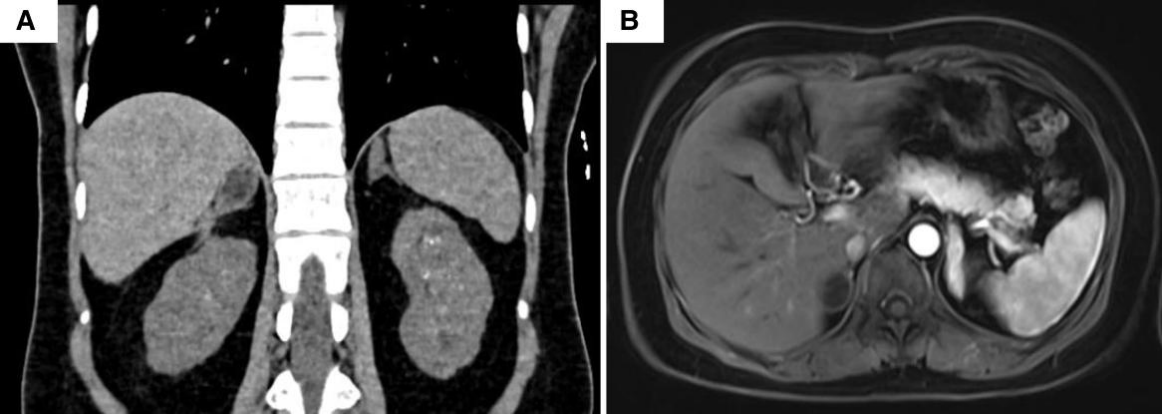
Coronal unenhanced computed tomography of the upper abdomen. The scan shows fat (HU: −27) containing myelolipoma in the right adrenal gland with hyperplasia of the limbs, hyperplasia of the left adrenal gland, medullary nephrocalcinosis in both the kidneys with a few small medullary cysts in the left kidney (A), and axial contrast enhanced T1-weighted fluid-attenuated inversion recovery sequence magnetic resonance imaging showing a fat-containing lesion in the right adrenal gland, suggestive of myelolipoma (2.3 × 1.7 × 2.6 cm), and homogenously enhancing hyperplastic left adrenal gland.

Ultrasound follow-up revealed a gradual reduction in the size of the right adrenal lesion over the 2 years. Computed tomography and magnetic resonance imaging at the last follow-up (2.5 years later) revealed a myelolipoma with hyperplastic limbs of right adrenal and hyperplastic left adrenal gland (Fig. 2). The imaging also unveiled medullary nephrocalcinosis and a few small renal cysts. The patient had well-controlled BP on oral dexamethasone (2 mg per day) with normal direct renin concentration and blood steroid profile except for a slightly elevated 11-deoxycorticosterone (Table 1).

## Discussion

GCRS is a rare disorder with fewer than 50 cases reported [2]. In this report, we present the first genetically proven case of GCRS from India. The prevalence of the disease might have been underestimated because of limited recognition of the condition. As observed in our patient, despite being diagnosed with hypertension at age 17 years, the confirmation of GCRS came a decade later when she experienced hypokalemic quadriparesis. Notably, presentation with such overt manifestations may only represent the tip of the iceberg of this disease, with a larger proportion of patients going undiagnosed or misdiagnosed. As observed in the affected sister, the manifestations can be as mild as acne resistant to topical therapy. The recognition of milder manifestations of GCRS has been increasing [4]. The delayed diagnosis of GCRS, even as late as the sixth decade, despite hyperandrogenism from puberty, has been reported [5]. In the French MUTA-GR study, heterozygous *NR3C1* variants were reported in approximately 5% of patients with bilateral adrenal hyperplasia, modestly elevated urinary free cortisol, incomplete cortisol suppression on a 1-mg dexamethasone suppression test, and lower aldosterone levels [6].

The presentation of GCRS is highly variable, encompassing a spectrum from severe manifestations such as atypical genitalia in 46 XX, hypertensive encephalopathy, and/or hypokalemic paralysis (severe manifestations) to milder manifestations including mild hypertension, acne, and/or hirsutism [2]. Our patient exhibited an intermediate phenotype characterized by moderate hypertension, acne, and hirsutism. These manifestations responded well to high-dose oral dexamethasone, whereas infertility management required ovulation induction, ultimately resulting in successful conception. However, hypokalemic quadriparesis was a severe manifestation that could have been precipitated by acute gastroenteritis. Hypokalemia in a young hypertensive patient should raise suspicion of mineralocorticoid excess, prompting further evaluation [7]. Low renin and low aldosterone hypertension should trigger an evaluation with a dexamethasone suppression cortisol test, not only to exclude endogenous CS but also GCRS. Excess mineralocorticoid activity, resulting from the spillover effect of cortisol on mineralocorticoid receptors because of saturation of 11β-hydroxysteroid dehydrogenase 2 in the kidneys and/or increased production of deoxycorticosterone in the zona fasciculata under ACTH drive, leads to hypertension and hypokalemia with suppression of renin and aldosterone [8].

To our knowledge, this is the first report of hypertension management during pregnancy in a patient with GCRS. Managing GCRS during pregnancy poses challenges. Although hydrocortisone is the safest glucocorticoid during pregnancy, it does not offer benefits for managing hypertension in GCRS because of its mineralocorticoid activity. Hence, fluorinated corticosteroids (dexamethasone, betamethasone) or prednisolone are preferred. The concentration of prednisolone in fetal circulation is approximately 10 times lower than that in maternal circulation because of its inactivation by 11β-hydroxysteroid dehydrogenase 2 and active retrograde transport by P-glycoprotein. Dexamethasone and betamethasone freely cross the placenta to the fetal circulation because they are not inactivated by 11β-hydroxysteroid dehydrogenase 2; it has a fetal:maternal concentration gradient of approximately 0.37 for betamethasone but with approximately 2-fold longer fetal half-life [9]. Hence, we considered changing from dexamethasone to prednisolone to minimize the potential teratogenicity. Notably, despite the replacement with equivalent doses of prednisolone, there was a resurgence of hypertension and hypokalemia, necessitating the resumption of antihypertensive and potassium supplements. This could be attributed to less effective ACTH suppression resulting from the shorter duration of action of prednisolone compared with dexamethasone. Besides, a contribution from additional ACTH production under the placental corticotropin-releasing hormone drive may be considered [10].

This is also the first reported case of adrenal hemorrhage in GCRS. The hemorrhage was spontaneous and unilateral (right adrenal) and occurred in the third trimester of pregnancy. However, the mechanisms underlying adrenal hemorrhage in this patient remain unclear. As revealed by the follow-up imaging, the hemorrhage occurred in the hyperplastic right adrenal gland with a myelolipoma. The increased occurrence of adrenal myelolipomas in patients with ACTH-driven adrenal hyperplasia is a well-described entity [11], often bilateral, and has also been reported in a case of GCRS [3]. Histological evidence of hemorrhage on cut surface is reported in 19%, whereas spontaneous rupture is reported in 4.5% adrenal myelolipoma cases [12]. Adrenal hemorrhage is a common entity in neonates, who have much larger adrenal glands than adults [13]. Pregnancy is associated with hypertrophy of the adrenal glands [14]. Spontaneous adrenal hemorrhage during pregnancy is uncommon and often bilateral [15]. Increased circulatory demand by the hyperplastic gland in the background of slow, singular venous drainage may predispose to hemorrhage [8]. In our patient, 1 or more of these 3 factors might have contributed to spontaneous adrenal hemorrhage. Although uncontrolled hypertension is often a triggering feature for adrenal hemorrhage, the normal BP at the previous visit (a week ago) and the presentation negate such a possibility.

Interestingly, our patient had medullary nephrocalcinosis and a few renal medullary cysts, which may be attributed to chronic kaliopenic nephropathy [16]. Another interesting point for discussion was the choice of glucocorticoid when the patient presented with a large adrenal hemorrhage in the third trimester. Although adrenal hemorrhage raised concerns for primary adrenal insufficiency [15], in which hydrocortisone is the ideal choice, betamethasone was chosen because the hemorrhage was unilateral and the fetus was borderline preterm.

Here, we report the first case of genetically proven GCRS from India with a novel likely pathogenic missense *NR3C1* variant in the homozygous state. Approximately 31 *NR3C1* loss-of-function mutations have been reported in patients with GCRS. Several functional studies have been proposed to assess the binding of the mutated protein (receptor) to glucocorticoids. However, because of limited resources, we were unable to perform functional studies to prove the variant's pathogenicity. Nevertheless, the low minor allele frequency, its deleterious nature in in silico analysis and segregation analysis with asymptomatic parents being heterozygous for the variant, and the affected sister being homozygous for the variant classifies the variant as pathogenic in the homozygous state. Interestingly, the variant was homozygous in our patient with autosomal recessive inheritance, which is rarer than autosomal dominant inheritance of the disease [2].

Here, we report the first case of spontaneous adrenal hemorrhage in a pregnant woman with GCRS. Indeed, this is also the first case of genetically proven GCRS from India with a novel likely pathogenic missense *NR3C1* variant in the homozygous state. The case illustrates the unique challenges of managing GCRS during pregnancy.

## Learning Points

- In this case report, we present the first case of spontaneous adrenal hemorrhage in a pregnant woman with glucocorticoid resistance syndrome (GCRS).
- This is also the first case of genetically proven GCRS from India with a novel likely pathogenic missense *NR3C1* variant in the homozygous state.
- The case illustrates the unique challenges of managing GCRS during pregnancy, which may have implications in managing other patients with this rare syndrome.

## Contributors

All authors made individual contributions to authorship. V.J., V.S., and M.K. were responsible for data collection and analysis and manuscript preparation. A.L., V.S., and T.B. were involved in the diagnosis and management of this patient. M.K. was responsible for manuscript submission. All authors reviewed and approved the final draft.

## Data Availability

Original data generated and analyzed during this study are included in this published article.

## Abbreviations

BP: blood pressure
CS: Cushing syndrome
GCRS: glucocorticoid resistance syndrome
